# Metabolic reprogramming in chronic kidney disease-cardiovascular disease comorbidity: from molecular mechanisms to therapeutic strategies

**DOI:** 10.3389/fphar.2026.1801502

**Published:** 2026-07-09

**Authors:** Xiang-jing Chai, Wei Gong, Wei Chen, Dong-yu Min, Yang Wang, Le Guan

**Affiliations:** 1 Liaoning University of Traditional Chinese Medicine, Shenyang, China; 2 Affiliated Hospital of Liaoning University of Traditional Chinese Medicine, Shenyang, China; 3 Postdoctoral Research Station, China Academy of Chinese Medical Sciences, Beijing, China

**Keywords:** cardiovascular disease, chronic kidney disease, gut microbiota, inflammation, metabolic reprogramming, mitochondrial dysfunction, SGLT2 inhibitors, uremic toxins

## Abstract

Chronic kidney disease (CKD) and cardiovascular disease are bidirectionally linked, with metabolic reprogramming as a central pathophysiological nexus. This review examines metabolic disturbances underlying CKD–CVD comorbidity, including energy metabolism disorders, mitochondrial dysfunction, lipid and glucose dysregulation, and mineral metabolism abnormalities. The gut microbiota–metabolic axis contributes through depletion of short-chain fatty acids, accumulation of trimethylamine N-oxide, and production of uremic toxins. These changes compromise intestinal barrier integrity and sustain systemic inflammation. Downstream consequences include oxidative stress, NLRP3 inflammasome activation, cardiac and renal fibrosis, vascular calcification, and endothelial dysfunction. We critically distinguish validated interventions from speculative strategies. SGLT2 inhibitors and GLP-1 receptor agonists have established cardiorenal protection in large randomized trials, with effects extending beyond glycemic control. By contrast, gut microbiota modulation, mitochondria-targeted therapies, and several anti-inflammatory and anti-fibrotic strategies remain at the preclinical or early-phase clinical stage, and a number of mechanistically attractive interventions have failed in confirmatory trials. We also outline areas of contradictory evidence and unresolved questions. Future directions include multi-omics integration, metabolic phenotype-based stratification, and rational drug development targeting central metabolic nodes. The review provides mechanistic insights into metabolic–immune–inflammatory crosstalk in CKD–CVD and highlights priorities for translation.

## 1 Introduction

CKD and CVD are leading public health problems worldwide. Global CKD prevalence is estimated at 9.1%, affecting more than 850 million people (Global and regional, 2020). The two diseases are tightly linked. In a cohort of over 1.1 million adults, patients with eGFR <15 mL/min/1.73 m^2^ had a 5.9-fold higher all-cause mortality and a 3.4-fold higher cardiovascular event risk than those with eGFR ≥60 mL/min/1.73 m^2^ (Go et al., 2004). CKD is now recognized as an independent CVD risk factor, and most CKD patients die from cardiovascular causes before progressing to end-stage renal disease (Sarnak et al., 2003).

Conventional risk factor control is insufficient in this population. Pooled community-based data confirm that eGFR <60 mL/min/1.73 m^2^ independently predicts CVD and all-cause mortality after adjustment for traditional risk factors (Weiner et al., 2004). Lipid-lowering and intensive glycemic control reduce, but do not eliminate, the excess risk. This residual risk has been attributed to CKD-specific pathways: uremic toxin accumulation, chronic low-grade inflammation, mineral metabolism disorders, and gut dysbiosis (Gansevoort et al., 2013). Identifying tractable mediators within these pathways is a central goal of current research.

Metabolic reprogramming has emerged as a candidate unifying framework. The term refers to systematic, disease-driven changes in cellular and systemic metabolism. It has been applied to both organ-specific injury and bidirectional crosstalk in CKD (Zhu et al., 2022). The framework extends the “common soil” hypothesis, which originally linked diabetes and atherosclerosis through shared genetic and environmental antecedents (Stern, 1995). In this view, metabolic abnormalities are not merely downstream consequences. They constitute a shared pathophysiological substrate. For example, indoxyl sulfate and p-cresyl sulfate induce vascular calcification through inflammation and coagulation pathways, providing one mechanistic link between renal solute retention and cardiovascular injury (Opdebeeck et al., 2019).

The evidence base for this framework is, however, heterogeneous. Some pathways are supported by large randomized outcome trials. Others rest mainly on preclinical models or biomarker-level human data. The DAPA-CKD trial, for instance, showed that dapagliflozin reduced the composite renal endpoint by 39% and cardiovascular death or heart failure hospitalization by 29% in CKD patients, independent of diabetes status (Heerspink et al., 2020). This and similar trials validate metabolic intervention as a clinically meaningful strategy. By contrast, gut microbiota modulation, mitochondria-targeted antioxidants, and several anti-fibrotic and anti-inflammatory programs remain investigational, and several mechanistically attractive interventions have failed in confirmatory trials. A clear-eyed appraisal that distinguishes validated therapies from speculative strategies, and human evidence from animal mechanistic data, is therefore essential when navigating this literature.

This review summarizes metabolic reprogramming in CKD–CVD comorbidity with explicit attention to evidence quality. We address: (1) the epidemiology and clinical features of CKD–CVD comorbidity; (2) energy metabolism disorders and mitochondrial dysfunction; (3) key signaling pathways and molecular mechanisms; (4) the gut microbiota–metabolism axis; and (5) systemic consequences of metabolic abnormalities. Throughout, we indicate whether findings derive from human trials, observational cohorts, or preclinical models; we differentiate established from emerging therapies; and we highlight contradictory or unresolved data where they exist.

## 2 Epidemiology and clinical features of CKD–CVD comorbidity

### 2.1 Epidemiological evidence

#### 2.1.1 Cardiovascular risk in CKD patients

**FIGURE 1:**
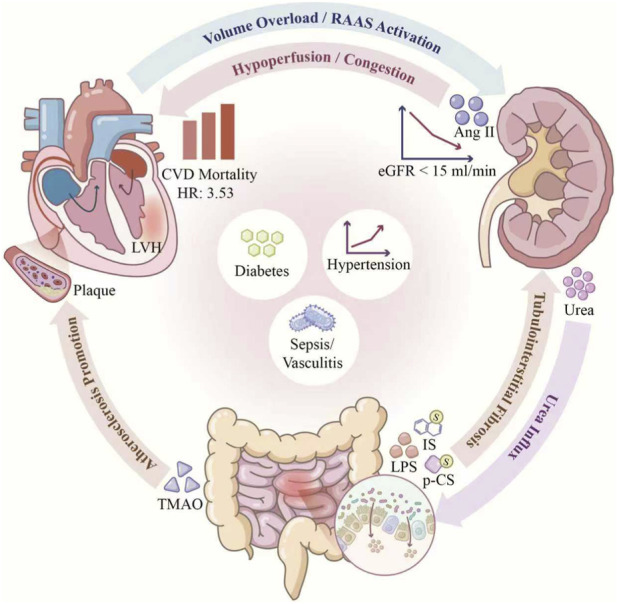
Overview of the heart–kidney–gut axis in CKD–CVD comorbidity. CKD and CVD drive each other through bidirectional hemodynamic, neurohormonal, and metabolic pathways. Cardiac dysfunction reduces renal perfusion through volume overload and RAAS activation (upper blue arrow), while renal failure damages the myocardium through hypoperfusion and venous congestion (upper pink arrow). Cardiovascular mortality rises progressively with declining renal function, reaching a hazard ratio of 3.53 at eGFR <15 mL/min/1.73 m^2^. Angiotensin II (Ang II)-mediated tubular injury, left ventricular hypertrophy (LVH), and atherosclerotic plaque formation constitute the structural substrate of comorbidity. The central panel depicts systemic conditions (diabetes, hypertension, sepsis/vasculitis) that simultaneously affect both organs, corresponding to Type V cardiorenal syndrome. The lower section illustrates the gut as a systemic amplifier: trimethylamine N-oxide (TMAO) generated by gut microbial metabolism promotes atherosclerosis; lipopolysaccharide (LPS), indoxyl sulfate (IS), and p-cresyl sulfate (p-CS) translocate across a “leaky” intestinal barrier into the circulation, aggravating tubulointerstitial fibrosis and sustaining systemic inflammation. Abbreviations: CKD, chronic kidney disease; CVD, cardiovascular disease; RAAS, renin–angiotensin–aldosterone system; Ang II, angiotensin II; eGFR, estimated glomerular filtration rate; HR, hazard ratio; LVH, left ventricular hypertrophy; TMAO, trimethylamine N-oxide; LPS, lipopolysaccharide; IS, indoxyl sulfate; p-CS, p-cresyl sulfate.

CVD is the leading cause of morbidity and mortality in CKD. The CKD Prognosis Consortium meta-analysis pooled data from over 1.2 million individuals (Matsushita et al., 2010). Cardiovascular mortality increased progressively with declining eGFR (HR 1.22, 1.69, and 3.53 at eGFR 75, 45, and 15 mL/min/1.73 m^2^, respectively) (Figure 1). The cardiovascular gradient was steeper than that for all-cause mortality, indicating that CKD confers cardiovascular-specific risk beyond general frailty.

The CKD-JAC cohort (n = 2,966) confirmed this gradient in a Japanese population. Cardiovascular event rates rose from 11.9 to 39.4 per 1,000 person-years across eGFR strata from 45 to 59 to <15 mL/min/1.73 m^2^. Patients in stage G5 had a 3.16-fold higher event risk than those in G3a (95% CI 1.28–7.76) (Tanaka et al., 2017). In dialysis patients, US Renal Data System data indicate that cardiovascular mortality is 10- to 30-fold higher than in age-matched controls.

#### 2.1.2 Renal risk in CVD patients

CKD is highly prevalent in heart failure. A pooled analysis of 57 studies and over 1 million patients found a CKD prevalence of approximately 32% (Damman et al., 2014). Moderate impairment (eGFR 30–59 mL/min/1.73 m^2^) conferred a 1.59-fold mortality risk. Severe impairment (eGFR <30 mL/min/1.73 m^2^) conferred a 2.17-fold risk (Damman et al., 2014).

CKD prevalence is high across all heart failure phenotypes. In a Swedish registry of 40,230 patients, CKD was present in 56% of HFpEF, 48% of HFmrEF, and 45% of HFrEF cases (Löfman et al., 2017). Coexistent CKD nearly doubled 1-year mortality in each subgroup (all P < 0.001).

#### 2.1.3 CKD staging and cardiovascular risk gradient

The KDIGO 2024 guideline stages CKD by combining eGFR with albuminuria categories (KDIGO, 2024 Clinical Practice Guideline, 2024). Albuminuria predicts cardiovascular events independently of filtration even at eGFR >60 mL/min/1.73 m^2^. It reflects glomerular endothelial dysfunction, podocyte injury, and systemic inflammatory activation. Patients with CKD G1–G2 and albuminuria ≥30 mg/g have cardiovascular event rates comparable to those with G3a without albuminuria (Matsushita et al., 2010; KDIGO, 2024 Clinical Practice Guideline, 2024). In a longitudinal cohort of 27,998 CKD patients, most died from cardiovascular causes before reaching ESRD ((Keith et al., 2004)). CV risk reduction must therefore begin at the time of CKD diagnosis, not at the threshold of dialysis dependence.

### 2.2 Clinical phenotypic features

#### 2.2.1 Classification of cardiorenal syndrome

Cardiorenal syndrome (CRS) refers to acute or chronic dysfunction of one organ inducing dysfunction of the other. The five-type classification first proposed in 2008 (Ronco et al., 2008) was subsequently refined in an AHA scientific statement (Rangas et al., 2019). Although this review focuses on chronic interactions, the full framework is presented because acute events (Types I and III) frequently initiate or accelerate chronic metabolic reprogramming.

Cardiorenal syndrome (CRS) refers to acute or chronic dysfunction of one organ inducing dysfunction of the other. The widely cited five-type classification distinguishes acute (Type I) and chronic (Type II) cardiorenal forms, acute (Type III) and chronic (Type IV) renocardiac forms, and a secondary form (Type V) in which a systemic condition such as diabetes, amyloidosis, vasculitis, or sepsis affects both organs simultaneously (Ronco et al., 2008; Rangas et al., 2019). Because this review focuses on chronic interactions, Types II and IV are most directly relevant, although acute events (Types I and III) frequently initiate or accelerate chronic metabolic reprogramming (Rosner and Okusa, 2006). The five categories are heuristic descriptors of temporal sequence rather than distinct mechanistic entities. Many patients display features of multiple types simultaneously, and the classification has not been validated against tissue-level mechanistic data or therapeutic responsiveness. It is best regarded as a communication framework rather than a pathophysiological taxonomy.

Critical caveat. The CRS classification provides clinical utility but should be interpreted cautiously. The five categories are heuristic descriptors of temporal sequence rather than distinct mechanistic entities. Many patients display features of multiple types simultaneously. The classification has not been validated against tissue-level mechanistic data or therapeutic responsiveness, and is best regarded as a communication framework rather than a pathophysiological taxonomy.

#### 2.2.2 Specific clinical manifestations

Uremic cardiomyopathy manifests as left ventricular hypertrophy, diastolic dysfunction, and myocardial fibrosis. FGF23 has been identified as a putative driver. In murine models, FGF23 directly induces cardiomyocyte hypertrophy through FGFR4-mediated calcineurin–NFAT signaling (Faul et al., 2011). Whether FGF23 is a causal mediator or a biomarker of cumulative phosphate burden in humans remains debated. FGF23-lowering interventions have not yet demonstrated cardiovascular benefit in randomized trials.

Vascular calcification is more pronounced in CKD than in the general population. The CRIC study identified lower eGFR, higher albuminuria, elevated serum phosphate, and increased FGF23 as independent predictors of coronary artery calcification progression (Bundy et al., 2018).

Sudden cardiac death and arrhythmias disproportionately burden ESRD patients. Sudden cardiac death accounts for over 25% of dialysis deaths, mediated by electrolyte imbalances, autonomic dysfunction, structural remodeling, and intradialytic hypotension (Genovesi et al., 2021).

#### 2.2.3 Prognostic assessment

Worsening renal function (WRF) carries context-dependent prognostic significance. WRF during effective decongestion, in the absence of residual congestion, does not portend adverse outcomes. WRF accompanied by persistent congestion, however, is associated with a 2.44-fold higher risk of death or heart failure rehospitalization (Metra et al., 2012). This distinction is clinically important. Not all eGFR drops during heart failure therapy reflect true renal injury, and the difference should guide treatment continuation versus dose adjustment.

Biomarker performance in CKD–CVD remains an active area of refinement. Cystatin C, NGAL, KIM-1, and IL-18 have been proposed for early AKI diagnosis and risk stratification (Coca et al., 2008). Their incremental clinical utility over conventional parameters has been variable across studies. Uniform thresholds for clinical decision-making are not yet established. NT-proBNP and high-sensitivity troponin have higher baseline values in CKD due to reduced renal clearance, requiring CKD-specific cutoffs to avoid misdiagnosis (House et al., 2019).

### 2.3 Pathophysiological mechanisms and treatment strategies

#### 2.3.1 Common pathophysiological mechanisms

The pathophysiological mechanisms of CKD–CVD comorbidity converge on metabolic reprogramming. Hemodynamic compromise drives mitochondrial hypoxic stress and substrate switching. Neurohormonal activation promotes gluconeogenesis, lipid accumulation, and pro-inflammatory cytokine production. The resulting inflammation–metabolism crosstalk amplifies oxidative stress and fibrogenesis. These molecular substrates are elaborated in subsequent sections.

The AHA scientific statement summarizes three mechanistic domains (Rangas et al., 2019):

##### 2.3.1.1 Hemodynamic

Low cardiac output reduces renal perfusion. Venous congestion elevates renal venous pressure, reducing the transglomerular pressure gradient. Increased intra-abdominal pressure further compromises perfusion.

##### 2.3.1.2 Neurohormonal

RAAS and sympathetic nervous system overactivation drive sodium and water retention, myocardial remodeling, and elevated afterload.

##### 2.3.1.3 Inflammatory and metabolic

Pro-inflammatory cytokines accelerate cardiac and renal fibrosis. Uremic toxins promote vascular aging. Mineral metabolism abnormalities drive vascular calcification.

#### 2.3.2 Treatment strategies

##### 2.3.2.1 Diuretics

Diuretics remain the cornerstone of congestion management (Mullens et al., 2019). Diuretic resistance is common in CKD; higher loop diuretic doses or combined thiazide therapy are often required. Loop diuretics, however, activate RAAS and sympathetic pathways that aggravate insulin resistance and aldosterone-driven fibrogenesis. RAAS inhibitors counteract this by attenuating angiotensin II–driven mitochondrial ROS, TGF-β signaling, and tubular epithelial metabolic reprogramming. They confer metabolic benefits beyond hemodynamic effects.

SGLT2 inhibitors—the most robustly validated metabolic intervention in CKD–CVD. Three large randomized trials have established cardiorenal protection across the CKD–CVD spectrum:

EMPA-REG OUTCOME (n = 7,020, type 2 diabetes with established CVD): empagliflozin reduced cardiovascular death by 38%, heart failure hospitalization by 35%, and all-cause mortality by 32% (Zinman et al., 2015).

DAPA-CKD (n = 4,304, eGFR 25–75 mL/min/1.73 m^2^): dapagliflozin reduced the renal composite endpoint by 39% and cardiovascular death or heart failure hospitalization by 29%, with benefits independent of diabetes status (Heerspink et al., 2020).

Pooled meta-analysis of five heart failure trials (DAPA-HF, EMPEROR-Reduced, EMPEROR-Preserved, DELIVER, SOLOIST-WHF; n = 21,947): SGLT2 inhibitors reduced the composite endpoint of cardiovascular death or heart failure hospitalization by 23%, cardiovascular death by 13%, first heart failure hospitalization by 28%, and all-cause mortality by 8%. Benefits were consistent across all ejection fraction phenotypes and glycemic states (Vaduganathan et al., 2022).

These trial-level data place SGLT2 inhibitors among the few interventions with confirmed mortality benefit in CKD–CVD. In contrast, the many mechanistically attractive but clinically unproven strategies discussed elsewhere in this review remain at the preclinical or early-phase stage. This distinction will be maintained throughout subsequent sections to keep the boundary between established and investigational therapies clear to the reader.

## 3 Metabolic dysfunction

The shared pathophysiological domains identified in Section 2 — bioenergetic stress, neurohormonal activation, and inflammation—converge on disordered cellular metabolism. This section examines six interconnected metabolic axes that link CKD to CVD: energy and mitochondrial dysfunction, lipid abnormalities, glucose dysregulation, amino acid disturbance, mineral imbalance, and nucleotide metabolism. A consistent theme is that mechanistic insight derives predominantly from rodent or cell-based systems, while human evidence is largely cross-sectional and biomarker-level. Few interventional confirmations exist outside the SGLT2 inhibitor class.

### 3.1 Energy metabolism disorders

#### 3.1.1 Mitochondrial dysfunction

**FIGURE 2:**
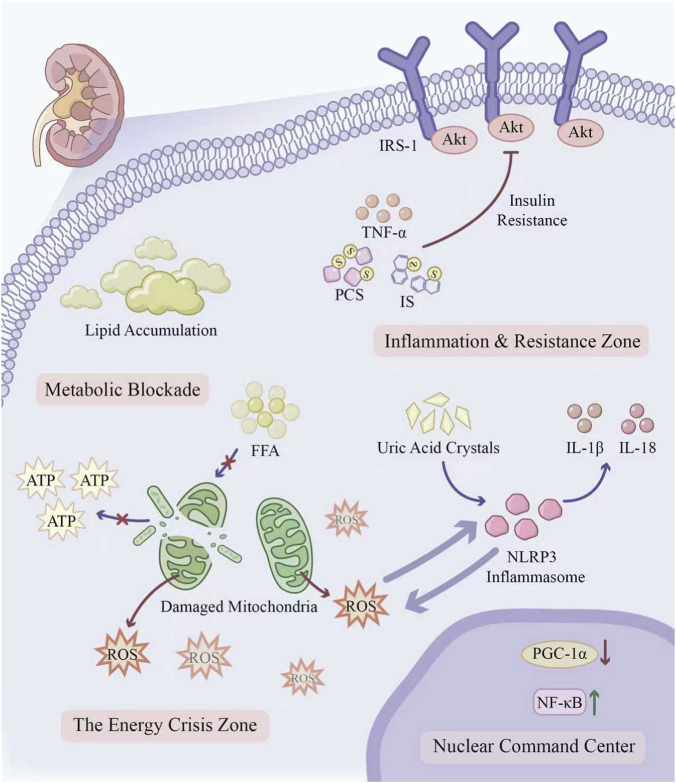
Key nodes of metabolic reprogramming in renal tubular epithelial cells. This schematic of a renal proximal tubular epithelial cell illustrates four interconnected zones of metabolic pathology in CKD–CVD comorbidity (Global and regional, 2020). Inflammation and Insulin Resistance Zone: TNF-α, p-CS, and IS promote serine phosphorylation of IRS-1 and inhibit downstream Akt signaling, producing insulin resistance (Go et al., 2004). Metabolic Blockade Zone: Free fatty acid (FFA) uptake into mitochondria is impaired, resulting in cytoplasmic lipid accumulation (Sarnak et al., 2003). Energy Crisis Zone: Damaged mitochondria fail to generate adequate ATP and leak reactive oxygen species (ROS), establishing a self-amplifying “ROS-induced ROS release” loop. ROS—together with uric acid crystals—activate the NLRP3 inflammasome, catalyzing maturation of IL-1β and IL-18 and triggering pyroptosis (Weiner et al., 2004). Nuclear Command Center: The master regulator of mitochondrial biogenesis PGC-1α is downregulated, while the pro-inflammatory transcription factor NF-κB is activated, forming the core circuit of metabolic–immune dysregulation. The figure integrates mechanisms discussed in Sections 3.1–3.3 (metabolic reprogramming) and 5.2.1 (NLRP3 inflammasome). Abbreviations: IRS-1, insulin receptor substrate 1; Akt, protein kinase B; TNF-α, tumor necrosis factor alpha; p-CS, p-cresyl sulfate; IS, indoxyl sulfate; FFA, free fatty acid; ATP, adenosine triphosphate; ROS, reactive oxygen species; NLRP3, NLR family pyrin domain containing 3; IL-1β, interleukin-1 beta; IL-18, interleukin-18; PGC-1α, peroxisome proliferator-activated receptor gamma coactivator 1-alpha; NF-κB, nuclear factor kappa B.

Mitochondria are the principal site of cellular energy generation. Renal proximal tubular cells and cardiomyocytes—both heavily dependent on oxidative phosphorylation—are particularly vulnerable to mitochondrial injury. Dysfunction in this organelle is a key node in CKD–CVD comorbidity (Figure 2).

##### 3.1.1.1 Impaired biogenesis

PGC-1α is the master regulator of mitochondrial biogenesis. It acts through NRF-1, NRF-2, and ERRα and is modulated by AMPK and SIRT1. PGC-1α is markedly downregulated in animal models of AKI, CKD, and heart failure, leading to reduced mitochondrial mass and impaired energy generation (Fontecha-Barriuso et al., 2020; Scarpulla, 2011).

##### 3.1.1.2 Reduced OXPHOS efficiency and ROS production

Inherited mitochondrial defects—including coenzyme Q10 biosynthesis abnormalities and mtDNA mutations—cause glomerular pathology in rare human disorders (Emma et al., 2016). In acquired disease, electrons leak from complex I (and to a lesser extent complex III) under conditions of high proton motive force, depleted CoQ pool, or elevated matrix NADH/NAD^+^ ratio, generating excess superoxide (Murphy, 2009). The “ROS-induced ROS release” mechanism describes a self-amplifying loop: mitochondrial permeability transition pore opening alters the redox state and accelerates ROS emission, which can propagate between mitochondria and trigger cell death (Zorov et al., 2014).

##### 3.1.1.3 Quality control

Mitochondrial homeostasis requires balance between biogenesis and degradation, mediated by mitophagy, the unfolded protein response, mitochondria-derived vesicles, and proteasomal pathways (Pickles et al., 2018; Tang et al., 2021).

##### 3.1.1.4 Proximal tubule-specific vulnerability

Renal proximal tubular cells depend almost exclusively on oxidative phosphorylation and possess minimal glycolytic reserve (Schaub et al., 2021). CKD progression is accompanied by a metabolic shift away from fatty acid oxidation toward glycolysis (Doke and Susztak, 2022). In a mouse model of obesity-induced CKD, proximal tubule-specific deletion of adenine nucleotide translocase 2 conferred renal protection by triggering metabolic reprogramming, indirectly establishing the central role of mitochondrial adenine nucleotide transport in tubular energy homeostasis (Permyakova et al., 2024).

##### 3.1.1.5 Pharmacokinetic implications

Tubular drug secretion depends on intact ATP supply. ABC efflux transporters use ATP directly. SLC influx transporters (OAT1/3, OCT2) operate differently—they rely on the electrochemical gradient maintained by Na^+^/K^+^-ATPase (Hoogstraten et al., 2023). Mitochondrial energetic failure impairs both routes. Drugs cleared by tubular secretion—including digoxin, methotrexate, and select antibiotics—therefore accumulate. This bioenergetic vulnerability adds to competitive OAT1/3 inhibition by uremic toxins. The combination creates a ‘dual pharmacokinetic vulnerability’ in CKD–CVD (Schaub et al., 2021; Doke and Susztak, 2022).

Much of the mitochondrial dysfunction framework in CKD–CVD derives from rodent ischemia–reperfusion or 5/6-nephrectomy models, with limited direct corroboration in human tissue beyond biopsy-based transcriptomic studies. Pharmacological translation has been disappointing: although mitochondria-targeted antioxidants (MitoQ, SS-31/elamipretide) and NAD^+^ precursors have shown encouraging biomarker effects in early-phase studies, trials of elamipretide (EMBRACE-STEMI, PROGRESS-HF) have failed to meet primary clinical endpoints. Whether the proximal tubular shift from FAO to glycolysis is causal or a downstream adaptation to injury remains unresolved.

#### 3.1.2 Substrate utilization impairment

Renal proximal tubular cells preferentially use β-oxidation of fatty acids under physiological conditions, because FAO yields more ATP than glycolysis (Gewin, 2021). After injury, FAO declines and lactate production rises. Capillary rarefaction following AKI causes intrarenal hypoxia, which propagates injury through tubular cells, fibroblasts, and inflammatory cells, ultimately producing tubulointerstitial fibrosis (Tanaka et al., 2014).

The strongest human evidence for FAO impairment comes from a transcriptomic analysis of 95 human kidney samples, in which inflammation and metabolism were the most dysregulated pathways in fibrotic kidneys. Defective FAO was characterized by reduced enzyme expression and intracellular lipid deposition. *In vitro* FAO inhibition reproduced ATP depletion, cell death, and dedifferentiation. In mice, restoration of fatty acid metabolism attenuated renal fibrosis (Kang et al., 2015). The clinical translation of FAO-restoring therapies, however, remains unproven.

### 3.2 Lipid metabolism abnormalities

Renal lipid accumulation correlates with declining kidney function in human diabetic nephropathy (Herman-Edelstein et al., 2014). The toxicity of accumulated lipid species reflects both quantity and composition. Loss of cardiolipin in the diabetic heart, for example, couples altered substrate utilization to mitochondrial dysfunction (Han et al., 2005).

CKD is characterized by a distinct dyslipidemia: elevated triglycerides, reduced HDL, and increased lipoprotein modification, particularly by carbamylation and oxidation (Vaziri, 2006). Carbamylated LDL impairs endothelial function *in vitro* (Speer et al., 2014), and protein carbamylation independently predicts mortality in dialysis patients (Koeth et al., 2013a). These uremia-specific lipoprotein modifications are mechanistically distinct from conventional dyslipidemia and may partly explain the limited efficacy of statins in late-stage CKD.

Ferroptosis—iron-dependent cell death driven by lipid peroxidation—is mechanistically rooted in CKD biology. Failure of glutathione peroxidase four to neutralize phospholipid hydroperoxides triggers lethal membrane peroxidation, amplified by the iron excess and antioxidant depletion characteristic of CKD. Synchronized renal tubular ferroptosis has been demonstrated in murine ischemia–reperfusion models (Linkermann et al., 2014). Direct human evidence for ferroptosis in CKD–CVD remains sparse. Most data derive from acute murine injury models; whether ferroptosis contributes meaningfully to chronic CKD progression in humans is unestablished. Ferroptosis-targeted therapies are not yet in advanced clinical development.

### 3.3 Glucose metabolism dysregulation

The uremic environment directly induces insulin resistance, demonstrated in classical clamp studies in dialysis patients (DeFronzo et al., 1981). Specific uremic solutes mediate this effect: p-cresyl sulfate promotes insulin resistance through inflammatory signaling (Koppe et al., 2013), and TNF-α contributes by promoting serine phosphorylation of IRS-1, inhibiting insulin receptor tyrosine kinase activity (Hotamisligil et al., 1996).

Advanced glycation end products (AGEs) accumulate in CKD due to impaired renal clearance and increased oxidative formation. AGE engagement of RAGE drives sustained NF-κB activation and perpetuates inflammatory and fibrotic signaling (Bierhaus et al., 2001). The AGE–RAGE axis links hyperglycemia and uremia to common downstream effectors. The relative contribution of AGE-driven injury versus uremia-specific mechanisms in non-diabetic CKD remains debated.

### 3.4 Amino acid metabolism disorders

Branched-chain amino acids function as both nutritional substrates and signaling molecules. Sustained BCAA elevation is linked to insulin resistance through mTORC1 hyperactivation and impaired insulin signaling (Lynch and Adams, 2014). In the Framingham Offspring cohort, prospective LC-MS/MS profiling of 2,422 normoglycemic participants showed that elevated isoleucine, leucine, valine, tyrosine, and phenylalanine predicted incident diabetes, with a >5-fold higher risk in the top quartile (Wang et al., 2011). Amino acid dysregulation thus precedes overt metabolic disease.

Asymmetric dimethylarginine (ADMA) accumulates in CKD due to reduced renal clearance and serves as an endogenous inhibitor of nitric oxide synthase (Böger, 2004). In a prospective cohort of 225 hemodialysis patients (median follow-up 33.4 months), plasma ADMA independently predicted all-cause mortality (HR 1.26, 95% CI 1.11–1.41) and cardiovascular events (HR 1.17, 95% CI 1.04–1.33) (Zoccali et al., 2001). ADMA represents one of the most robustly characterized links between CKD biochemistry and endothelial dysfunction in human studies.

### 3.5 Mineral metabolism disorders

Phosphate retention is a defining feature of advanced CKD and a major driver of vascular and skeletal pathology (Ritter and Slatopolsky, 2016). Hyperphosphatemia promotes vascular smooth muscle cell phenotypic transition: contractile markers (SM22α, smooth muscle α-actin) are downregulated, while osteogenic transcription factors (Cbfa1) and matrix proteins (osteopontin, osteocalcin) are upregulated (Steitz et al., 2001). The result is conversion of medial smooth muscle into a calcification-prone phenotype.

FGF23 and Klotho. FGF23 rises before parathyroid hormone and phosphate in early CKD (Isakova et al., 2011). Klotho deficiency drives vascular calcification in CKD models (Hu et al., 2011). In the CRIC cohort (n = 3,070), FGF23 levels were independently associated with left ventricular hypertrophy. In murine experiments, FGF23 induced cardiomyocyte hypertrophy through klotho-independent FGFR4–calcineurin–NFAT signaling, an effect blocked by FGF receptor inhibition (Faul et al., 2011).

#### 3.5.1 Critical caveat

As discussed in Section 2, FGF23 as a direct cardiovascular mediator should be interpreted with caution. The mechanistic data are compelling in animal models, but FGF23-lowering interventions—including cinacalcet in EVOLVE and phosphate binders in dedicated programs—have not produced consistent cardiovascular benefit in humans. Whether FGF23 is a modifiable target or a sensitive biomarker of cumulative phosphate burden remains unresolved.

### 3.6 Nucleotide metabolism disorders

Uric acid is the end product of purine catabolism and the dominant nucleotide-related metabolite in CKD–CVD (Jalal et al., 2013). An early prospective trial showed that allopurinol slowed eGFR decline and reduced cardiovascular risk in CKD patients (Goicoechea et al., 2010). Subsequent larger trials—CKD-FIX and PERL (febuxostat) — did not show meaningful slowing of renal function decline, raising the unresolved question of whether uric acid is a causal driver or a biomarker of disease. Routine urate-lowering as a renoprotective strategy outside symptomatic gout therefore lacks robust trial support.

#### 3.6.1 NAD^+^ homeostasis

NAD^+^ is essential for redox reactions and serves as substrate for sirtuins and PARP enzymes (Katsyuba et al., 2020). PGC-1α coordinates *de novo* NAD^+^ biosynthesis and renal recovery from injury. PGC-1α-deficient mice develop NAD^+^ precursor deficiency, lipid accumulation, and impaired functional recovery after renal ischemia. Exogenous nicotinamide ameliorates these abnormalities (Tran et al., 2016). NAD^+^ precursors are now in early-phase clinical trials in AKI and heart failure, but no large outcome trial has yet established benefit.

## 4 The role of the gut microbiota–metabolic axis in CKD–CVD comorbidity

Beyond the organ-level metabolic dysfunction described in Section 3, the gut microbiota constitutes a systemic amplifier of CKD–CVD pathology. A systematic review of 69 studies reported an association between gut dysbiosis and approximately 35% of cardiovascular-related mortality in CKD patients (Voroneanu et al., 2023). Dysbiosis is not merely a downstream consequence of declining renal function. It actively contributes to disease through three interacting mechanisms: altered microbial metabolite production, intestinal barrier disruption, and sustained systemic inflammation (Huang Y. et al., 2022).

### 4.1 Gut dysbiosis

#### 4.1.1 Microbiota characteristics in CKD patients

Both α- and β-diversity of the gut microbiota are significantly reduced in CKD (Amini Khiabani et al., 2023). At the phylum level, Firmicutes and Proteobacteria are enriched while Bacteroidetes are depleted (Tang et al., 2023). At the genus and species level, beneficial bacteria—including *Bifidobacterium*, *Lactobacillus*, and *Faecalibacterium prausnitzii*—are markedly decreased, while potentially pathogenic genera such as *E. coli* and *Enterococcus* expand (Altamura et al., 2023). In a systematic review, *Ruminococcus* and *Roseburia* showed discriminatory power between CKD patients and healthy controls (AUC 0.771 and 0.803, respectively); *Roseburia* depletion was most pronounced in end-stage kidney disease (P < 0.001). A microbiota-based model demonstrated excellent predictive power for diabetic nephropathy (AUC = 0.972) (Voroneanu et al., 2023).

#### 4.1.2 Microbiota changes in CVD patients

SCFA-producing bacteria—*Roseburia*, *Eubacterium*, and *Faecalibacterium*—are depleted in CVD patients (Altamura et al., 2023). Coronary artery disease is associated with enrichment of TMA-producing bacteria that convert dietary choline and L-carnitine to TMA, which is subsequently hepatically oxidized to TMAO (Koeth et al., 2013b).

#### 4.1.3 Synergistic dysbiosis in comorbid states

Gut dysbiosis in CKD–CVD comorbidity exceeds the severity observed in either condition alone (Caldarelli et al., 2023). In CKD, renal insufficiency promotes urease-producing bacterial overgrowth. Bacterial hydrolysis of urea releases ammonia, which disrupts intestinal pH and mucosal barrier integrity (Vaziri et al., 2013). This creates a self-reinforcing loop: dysbiosis worsens uremic toxin production, which further damages the barrier and promotes dysbiosis.

### 4.2 Microbial metabolites

#### 4.2.1 Short-chain fatty acid depletion

SCFAs—acetate, propionate, and butyrate—are produced by anaerobic fermentation of dietary fiber (Magliocca et al., 2022). They act through G protein-coupled receptors (GPR41, GPR43, GPR109 A) and HDAC inhibition (Mann et al., 2024). In CKD, fecal and plasma SCFA levels are significantly reduced, correlating with depletion of *F. prausnitzii* and *Roseburia* (Lambert et al., 2023).

The consequences are dual. First, SCFAs are the primary energy source for colonocytes; their loss impairs mucosal barrier function. Second, SCFAs suppress NF-κB signaling, promote regulatory T cell differentiation, and reduce IL-6, IL-1β, and TNF-α production (Arpaia et al., 2013). In a human cohort study, high dietary fiber intake was associated with decreased inflammation and all-cause mortality in CKD patients, an effect partially attributed to SCFAs (Krishnamurthy et al., 2012).

#### 4.2.2 TMAO elevation

Dietary choline and L-carnitine are converted by gut bacteria to TMA, then hepatically oxidized by FMO3 to TMAO. In a prospective study of 2,595 cardiac evaluation subjects, plasma L-carnitine predicted increased CVD risk and MACE only among those with concurrently high TMAO levels, establishing TMAO as a critical modifier (Koeth et al., 2013b). TMAO accumulates further in CKD due to reduced renal excretion. In a 5-year prospective cohort of 521 CKD patients (eGFR <60 mL/min/1.73 m^2^), median TMAO was 7.9 μmol/L versus 3.4 μmol/L in non-CKD controls. The highest TMAO quartile carried a 2.8-fold higher mortality risk. After adjustment for conventional risk factors, hsCRP, and eGFR, TMAO remained an independent predictor of 5-year mortality (HR 1.93, 95% CI 1.13–3.29) (Tang et al., 2015). TMAO also promotes arterial thrombosis by inducing endothelial tissue factor expression. In 2,989 stable CVD patients, TMAO associated with a 1.73-fold higher 3-year MACE risk (Witkowski et al., 2022).

A gut microbe-targeted CutC/D enzyme inhibitor reduced plasma TMAO and rescued diet-induced platelet hyperreactivity in murine models without apparent toxicity (Roberts et al., 2018).

The causal status of TMAO in humans remains contested. Several analyses have reported attenuated or null associations after adjustment for renal function, suggesting TMAO elevation may partly reflect—rather than cause—declining GFR. Virtually all mechanistic data derive from murine models with TMAO concentrations far exceeding human physiological levels. The CutC/D inhibitor approach remains preclinical, with no human outcome trial completed. TMAO should be regarded as a biologically plausible mediator with strong epidemiological but unconfirmed causal support.

#### 4.2.3 Uremic toxin accumulation

Indoxyl sulfate (IS) and p-cresyl sulfate (PCS) are protein-bound uremic toxins generated by intestinal bacterial metabolism of tryptophan and tyrosine. Their mechanisms of toxicity have been systematically reviewed (Vanholder et al., 2014). PCS promotes NADPH oxidase-mediated ROS generation in renal tubular cells (Watanabe et al., 2013). Both IS and PCS are independently associated with cardiovascular events and all-cause mortality in CKD patients (Lin et al., 2015).

IS and PCS are cleared renally via OAT1 (SLC22A6) and OAT3 (SLC22A8) on proximal tubular cells (Bush et al., 2020). As CKD advances, these transporters also mediate secretion of cardiovascular drugs (ACE inhibitors, furosemide). Accumulating IS and PCS compete directly with co-administered drugs for OAT1/3 binding, reducing drug secretion independently of GFR decline (Tan et al., 2023). PBPK modeling using the endogenous OAT1/3 biomarker 4-pyridoxic acid has confirmed that active secretion deteriorates faster than glomerular filtration in CKD (84). Furthermore, loss of renal OAT1 function causes systemic accumulation of over 40 microbiome-dependent metabolites (Granados et al., 2023). Compensatory upregulation of intestinal ABCG2 (BCRP) partially offsets this, but transporter-function-informed dosing strategies remain underutilized (Lowenstein and Nigam, 2021).

Despite compelling mechanistic data, IS and PCS remain pharmacologically intractable. The oral adsorbent AST-120, designed to reduce IS precursor absorption, failed to slow CKD progression in the EPPIC-1 and EPPIC-2 trials and showed no cardiovascular benefit. This raises the unresolved question of whether IS and PCS are causal mediators or sensitive biomarkers of advanced uremia.

### 4.3 Intestinal barrier dysfunction

#### 4.3.1 Increased intestinal permeability

The intestinal mucosal barrier comprises epithelial tight junctions, mucus layer, gut-associated lymphoid tissue, and microbiota (Turner, 2009). In rat CKD models (5/6 nephrectomy and adenine-induced), uremia disrupted tight junctions with decreased claudin-1, occludin, and ZO-1 protein expression despite preserved mRNA levels, suggesting post-translational dysregulation (Vaziri et al., 2012). Corresponding human evidence is limited to small case series and immunohistochemistry on dialysis colonic biopsies; large-scale tissue-level confirmation in CKD patients remains lacking.

*F. prausnitzii* depletion was confirmed in two independent CKD populations (Western: n = 283; Chinese: n = 75). *F. prausnitzii* supplementation attenuated CKD in mice via the butyrate–renal GPR43 axis. AAV-mediated GPR43 knockdown abolished this protective effect (Li et al., 2022).

#### 4.3.2 Endotoxemia

Barrier disruption allows LPS to enter the circulation. Dialysis patients show significantly elevated endotoxin levels (0.62 ± 0.37 vs. 0.11 ± 0.68 EU/mL), closely associated with inflammatory markers and cardiovascular risk (McIntyre et al., 2011). LPS activates TLR4, triggering NF-κB and MAPK cascades and inducing pro-inflammatory cytokines, chemokines, and adhesion molecules (Linh et al., 2022).

#### 4.3.3 Systemic inflammation exacerbation

Gut-derived LPS, peptidoglycan, and bacterial DNA activate pattern recognition receptors (TLRs and NLRs), sustaining systemic inflammation (Linh et al., 2022). Intestinal barrier dysfunction and gut dysbiosis form a mutually reinforcing cycle: dysbiosis depletes beneficial metabolites and increases harmful ones, further damaging the barrier; barrier damage worsens the intestinal microenvironment and promotes further dysbiosis (Anders et al., 2013). Disrupting this cycle is a primary rationale for microbiota-targeted therapeutic strategies, discussed in Section 6.

Direct human evidence for pharmacological intestinal barrier restoration in CKD remains absent. Most barrier disruption data derive from rat 5/6 nephrectomy and adenine-induced CKD models, with corresponding human evidence limited to small case series and immunohistochemistry on dialysis colonic biopsies. Although the conceptual rationale for “leaky gut” as a CKD–CVD amplifier is robust, no large RCT has yet demonstrated that restoring barrier integrity reduces hard cardiorenal endpoints. Probiotic, prebiotic, and fiber-based interventions have shown short-term improvements in biomarkers such as serum LPS and SCFA levels but have not progressed to outcome-level validation. The barrier-restoration paradigm should therefore be regarded as biologically plausible but clinically unproven.

## 5 Systemic effects of metabolic abnormalities

The metabolic disturbances and gut–microbiota dysregulation described in Sections 3, 4 do not remain organ-confined. They propagate through five interconnected systemic effectors—oxidative stress, chronic inflammation, fibrosis, vascular calcification, and endothelial dysfunction—that collectively define the structural and functional substrate of CKD–CVD comorbidity. These effectors operate as a network, with extensive cross-amplification between domains.

### 5.1 Oxidative stress

Oxidative stress reflects an imbalance between ROS generation and antioxidant defense. The contemporary view extends beyond simple redox imbalance to encompass dysregulated redox signaling affecting nearly all cellular processes.

#### 5.1.1 Increased ROS generation

Multiple sources contribute to excess ROS in metabolic disease. Mitochondrial electron leakage at complexes I and III generates superoxide, particularly under conditions of nutrient excess and low ATP demand that hyperpolarize the inner membrane potential (Bhargava and Schnellmann, 2017). NADPH oxidases (NOX2 and NOX4) are upregulated by angiotensin II, AGEs, and pro-inflammatory cytokines acting via PKC and NF-κB. NOX4 is especially relevant in the kidney, where it is highly expressed in glomerular podocytes and tubular epithelial cells (Nlandu Khodo et al., 2012). *In vitro*, indoxyl sulfate increases NOX4 activity and superoxide generation more than threefold in human proximal tubular (HK-2) cells, triggering mitochondrial depolarization and apoptosis at concentrations physiologically relevant to CKD (80).

Xanthine oxidase activity is elevated in hyperuricemia and generates O_2_^−^ and H_2_O_2_ during purine catabolism (Kimura et al., 2021). Uric acid further activates NOX, creating a feed-forward loop. Xanthine oxidase inhibitors reduce serum urate, lower urinary 8-OHdG, and modestly slow eGFR decline in early CKD (Jalal et al., 2013). Subsequent larger trials (CKD-FIX with allopurinol; PERL with febuxostat) have not confirmed clinically meaningful renoprotection, leaving uncertain whether xanthine oxidase inhibition is a true metabolic intervention or merely a urate-lowering adjunct in symptomatic gout.

Endothelial NOS uncoupling is a particularly damaging ROS source because it simultaneously eliminates protective NO production. Causes include BH4 oxidation, ADMA accumulation, and S-glutathionylation of the eNOS reductase domain (Förstermann et al., 2017). In CKD, ADMA accumulates due to reduced renal clearance and predicts cardiovascular mortality in dialysis patients (Böger, 2004). This represents a CKD-specific molecular link between uremic biochemistry and endothelial dysfunction.

#### 5.1.2 Impaired antioxidant defense

Antioxidant capacity declines progressively in metabolic disease. Nrf2 — the master regulator of cytoprotective gene transcription—undergoes adaptive dysregulation under sustained oxidative stress: early activation transitions to chronic burnout, with Nrf2 promoter methylation contributing to late-stage suppression (Ruiz et al., 2013). SOD activity is reduced in CKD, partly due to uremic toxin-mediated enzyme oxidation and zinc deficiency. Glutathione synthesis is also impaired, with reduced cysteine availability and increased GSSG efflux contributing to a low GSH/GSSG ratio (Moldogazieva et al., 2018).

Despite robust mechanistic support for antioxidant defense impairment, broad-spectrum antioxidant interventions have repeatedly failed in cardiovascular trials. Vitamin E showed no benefit (and possibly harm) in HOPE and HOPE-TOO. Vitamin C and β-carotene similarly failed across multiple endpoints. The Nrf2 activator bardoxolone methyl was halted in BEACON because of excess heart failure events. These failures suggest that indiscriminate scavenging may disrupt physiological redox signaling rather than restore it. Future strategies must be more granular—targeting specific ROS sources, subcellular compartments, or endogenous defense regulation.

#### 5.1.3 Accumulation of oxidative damage

Lipid peroxidation generates 4-hydroxynonenal and malondialdehyde, which form adducts with proteins and DNA and alter membrane fluidity (Moldogazieva et al., 2018). Oxidized LDL is central to atherosclerotic plaque biology. Protein carbonylation and 8-OHdG (a marker of DNA oxidative damage) correlate with CKD progression rate (Valavanidis et al., 2009). Oxidative damage to mtDNA establishes a self-perpetuating cycle: damaged mitochondria produce more ROS while losing oxidative phosphorylation capacity.

### 5.2 Inflammatory response

Chronic low-grade inflammation—termed “metaflammation” — links metabolic disorders to multi-organ injury. Unlike acute infectious inflammation, metaflammation is persistent, mild, and widespread.

#### 5.2.1 Chronic low-grade inflammatory state

Immune and metabolic regulation are tightly coupled at cellular and systemic levels. Their dysregulation underlies obesity, type 2 diabetes, and cardiovascular disease (Meijers et al., 2019). Adipose tissue is a major metaflammation source: hypertrophied adipocytes activate HIF-1α and ER stress; macrophages polarize from M2 to M1; and crown-like structures form around dying adipocytes.

CKD amplifies metaflammation through three mechanisms: impaired renal clearance of inflammatory mediators prolonging cytokine half-lives; uremic toxin-mediated AhR and NF-κB activation; and gut dysbiosis-derived LPS sustaining TLR4 signaling. Circulating IL-6 and TNF-α are 2–5-fold elevated in CKD patients without overt infection, as documented across multiple CKD cohorts including the seminal observational work of Stenvinkel and colleagues. This baseline activation independently predicts cardiovascular events and mortality (Lowenstein and Nigam, 2021).

The NLRP3 inflammasome integrates metabolic danger signals—saturated fatty acids, cholesterol crystals, urate crystals, glucose—and produces caspase-1-mediated IL-1β/IL-18 maturation and gasdermin D-driven pyroptosis (Figure 2) (Swanson et al., 2019).

#### 5.2.2 Inflammatory cytokine network

TNF-α activates IKK/NF-κB and JNK, promoting IRS-1 serine phosphorylation, blocking GLUT4 translocation, and downregulating adiponectin receptors (Cawthorn and Sethi, 2008). IL-6 drives hepatic CRP production via JAK/STAT3 and contributes to insulin resistance (Kang et al., 2019). CRP elevation has become a clinical marker of cardiovascular risk. IL-1β acts through MyD88/NF-κB; canakinumab (IL-1β blockade) reduced cardiovascular events in CANTOS ((Dinarello, 2011)). MCP-1/CCL2 mediates monocyte recruitment via CCR2 and is upregulated in obese adipose tissue and CKD kidneys.

While CANTOS established proof-of-concept that inflammation is a modifiable cardiovascular target, several caveats apply to CKD–CVD. Canakinumab’s absolute risk reduction was modest (∼0.3% per year) and came at the cost of increased fatal infections. The trial did not include dedicated renal endpoints. Broader anti-inflammatory programs targeting IL-6 (ZEUS, RESCUE) remain unproven. Translation to a population already burdened by uremia-associated immunoparesis demands caution.

##### 5.2.2.1 Inflammation-driven pharmacokinetic dysregulation

Two nuclear receptors regulate drug metabolism and biliary excretion: pregnane X receptor (PXR/NR1I2) and constitutive androstane receptor (CAR/NR1I3). They control CYP3A4, MDR1/P-gp, and MRP2/ABCC2 expression (Poudel et al., 2023). PXR and NF-κB act in mutual antagonism. Activated NF-κB p65 disrupts PXR DNA binding and suppresses target gene induction. In CKD–CVD, IL-6 reduces PXR expression. TNF-α attenuates CYP3A4 inducibility in primary hepatocyte systems. Bioinformatic evidence from COVID-19 pharmacotherapy supports this “inflammatory silencing” mechanism as a clinical driver of drug-induced hepatotoxicity (Huang J. et al., 2022). Gut dysbiosis further disrupts intestinal PXR activation by depleting endogenous ligands such as indole-3-propionic acid (Dutta et al., 2022). The clinical consequence is reduced drug clearance precisely in patients with the highest cardiovascular risk, providing a pharmacological rationale for anti-inflammatory therapy beyond its direct cardiovascular effects (Lowenstein and Nigam, 2021; Dutta et al., 2022).

#### 5.2.3 Inflammation–metabolism vicious cycle

Inflammation impairs insulin signaling by promoting IRS-1 serine phosphorylation and degradation, producing insulin resistance and compensatory hyperinsulinemia (Vanholder et al., 2018). Oxidative stress and inflammation reinforce each other: ROS activate NF-κB; activated immune cells produce more ROS (Morgan and Liu, 2011). Uremic toxins (p-cresyl sulfate, indoxyl sulfate) stimulate AhR and NF-κB and are poorly cleared by standard dialysis, sustaining chronic inflammation (Vanholder et al., 2018).

### 5.3 Fibrotic process

**FIGURE 3:**
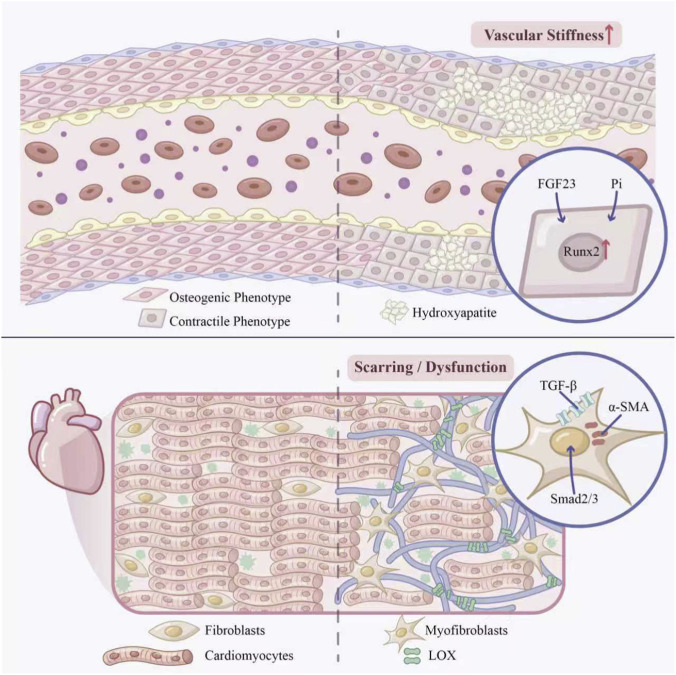
Structural remodeling in CKD–CVD: vascular calcification (upper) and cardiac fibrosis (lower). Upper panel—vascular calcification and arterial stiffness: In the hyperphosphatemic and FGF23-elevated milieu of CKD, medial vascular smooth muscle cells (VSMCs) undergo phenotypic transition from a contractile phenotype (loss of contractile markers including SM22α and α-SMA) to an osteogenic phenotype (upregulation of Runx2), ultimately depositing hydroxyapatite within the extracellular matrix. The result is Mönckeberg-type medial calcification and a marked increase in vascular stiffness. The inset depicts the core pathway by which inorganic phosphate (Pi) and FGF23 drive Runx2 upregulation through membrane signaling. Lower panel—cardiac fibrosis and dysfunction: TGF-β binds its receptor and phosphorylates the Smad2/3 complex, which translocates to the nucleus and activates target gene transcription. Quiescent fibroblasts are converted into α-SMA-expressing myofibroblasts that secrete type I and type III collagen. Lysyl oxidase (LOX) catalyzes cross-linking of collagen and elastin, generating an irreversible scar-like matrix that disrupts the functional alignment of cardiomyocytes and ultimately produces ventricular stiffening and diastolic dysfunction. Abbreviations: VSMC, vascular smooth muscle cell; Pi, inorganic phosphate; FGF23, fibroblast growth factor 23; Runx2, runt-related transcription factor 2; SM22α, smooth muscle protein 22-alpha; α-SMA, alpha-smooth muscle actin; TGF-β, transforming growth factor beta; Smad2/3, mothers against decapentaplegic homolog 2/3; LOX, lysyl oxidase.

Fibrosis is a common final pathway in CKD and CVD. It reflects excess ECM deposition that initially serves repair but becomes pathological when persistent or dysregulated (Wynn, 2008). Cardiac and renal fibrosis are the most clinically consequential manifestations, leading to heart failure and ESRD respectively (Figure 3).

#### 5.3.1 Cardiac and renal fibrosis

Cardiac fibrosis takes two forms: replacement fibrosis after cell loss, and reactive interstitial fibrosis without cell death. Quiescent fibroblasts are activated by angiotensin II, TGF-β, aldosterone, oxidative stress, and mechanical strain. Activated fibroblasts express α-SMA, transform into myofibroblasts, and secrete type I and III collagen (Travers et al., 2016). Failure of myofibroblast apoptosis after injury characterizes pathological fibrosis.

Renal fibrosis is the common pathway through which CKD progresses to ESRD regardless of initiating cause (Zeisberg and Neilson, 2010). Tubulointerstitial fibrosis correlates best with declining renal function. Snail1-driven partial epithelial-mesenchymal transition (partial EMT) of tubular cells is a key driver. In partial EMT, tubular cells acquire mesenchymal features without full epithelial loss, undergo cell cycle arrest, and produce profibrotic factors (Grande et al., 2015).

#### 5.3.2 TGF-β/smad pathway

TGF-β is the master fibrotic regulator. Latent TGF-β1 is activated and binds TβRII, which recruits TβRI to phosphorylate Smad2/3. The Smad2/3–Smad4 complex translocates to the nucleus and activates target gene transcription (Meng et al., 2016; Derynck and Zhang, 2003). Smad3 is particularly critical: its deficiency attenuates multiple fibrosis models (Lan, 2011). Smad7 antagonizes the pathway through receptor-binding competition, Smurf-mediated receptor degradation, and PP1-mediated dephosphorylation. TGF-β also signals through non-Smad pathways including MAPK, PI3K/Akt, and Rho-GTPases (Zhang, 2009).

Despite extensive mechanistic dissection, direct TGF-β inhibition has been notably difficult to translate. Pirfenidone—approved in idiopathic pulmonary fibrosis—has not shown consistent benefit in diabetic nephropathy. Anti-CTGF antibodies (pamrevlumab) have produced mixed results, and dedicated anti-TGF-β monoclonal programs in fibrotic kidney disease have been limited by on-target toxicity given TGF-β′s homeostatic immune and epithelial roles. The TGF-β/Smad axis should therefore be viewed as a robustly characterized but still pharmacologically intractable target.

#### 5.3.3 Extracellular matrix remodeling

ECM remodeling reflects imbalance between synthesis and degradation. Type I collagen accumulation is the major contributor to tissue stiffness (Weber et al., 2013). MMPs mediate degradation; TIMPs are upregulated during fibrosis, shifting the balance toward accumulation (Bonnans et al., 2014). Lysyl oxidase catalyzes lysine oxidative deamination on collagen and elastin, generating cross-links that increase mechanical strength and degradation resistance (Cox and Erler, 2011). LOX is induced by TGF-β, hypoxia, and oxidative stress, linking pro-fibrotic stimuli to ECM cross-linking. AGE-mediated non-enzymatic cross-linking provides an additional pathway under hyperglycemia.

### 5.4 Vascular calcification

Vascular calcification is now recognized as an active, cell-regulated osteochondrogenic process rather than passive degeneration. This paradigm shift implies it is potentially preventable or reversible.

#### 5.4.1 Calcium-phosphate deposition and calcification inhibitors

Hyperphosphatemia drives VSMC osteochondrogenic differentiation through PiT-1, while elevated calcium promotes apoptosis and matrix vesicle release (Shanahan et al., 2011; Hruska et al., 2008). Loss of calcification inhibitors plays a central role. Matrix Gla protein (MGP) requires vitamin K-dependent γ-carboxylation to bind calcium and inhibit BMP signaling. Pyrophosphate inhibits hydroxyapatite crystal formation, but is hydrolyzed by tissue non-specific alkaline phosphatase, which is upregulated in calcifying vessels (Villa et al., 2009). Other inhibitors include fetuin-A, osteopontin, and osteoprotegerin.

Translation of these mechanistic insights has been disappointing. Vitamin K supplementation trials (VitaK-CAC, K4Kidneys) have not reproducibly slowed vascular calcification in CKD. Sevelamer and lanthanum reduce phosphate but have not produced consistent calcification regression. Cinacalcet narrowly missed its primary endpoint in EVOLVE. The osteogenic paradigm of vascular calcification remains better established mechanistically than therapeutically.

#### 5.4.2 VSMC transdifferentiation and vascular stiffness

VSMCs lose contractile markers (SM22α, smooth muscle myosin heavy chain, α-SMA) and acquire osteoblast- or chondrocyte-like phenotypes. Runx2 is the central transcription factor driving this conversion, activating osteocalcin, alkaline phosphatase, and other mineralization genes (Figure 3, upper panel). BMP-2 and BMP-4 induce osteogenic differentiation through Smad1/5/8, while Wnt/β-catenin signaling, inflammation, oxidative stress, and uremic toxins all converge to promote transdifferentiation (Giachelli, 2004; Boström et al., 2011; Libby, 2002).

The hemodynamic consequence is increased arterial stiffness, measured clinically by pulse wave velocity, which independently predicts cardiovascular events and mortality with particular strength in CKD and diabetic populations (Vlachopoulos et al., 2010). Medial calcification (Mönckeberg’s arteriosclerosis) markedly reduces vascular compliance, producing elevated systolic pressure with increased afterload, reduced diastolic pressure with compromised coronary perfusion, and widened pulse pressure (London et al., 2003). VSMC transdifferentiation and arterial stiffness are thus mechanistically and clinically inseparable expressions of the same osteochondrogenic process.

### 5.5 Endothelial dysfunction

Endothelial dysfunction is an early event in metabolic cardiovascular complications, preceding structural lesions by years. It serves as a common initiator for both atherosclerosis and microvascular disease.

#### 5.5.1 Endothelial cell activation

Activated endothelial cells upregulate VCAM-1 and ICAM-1 in an NF-κB-dependent manner, recruiting leukocytes and initiating atherogenesis (Cook-Mills et al., 2011). They acquire a prothrombotic phenotype with increased tissue factor expression (Steffel et al., 2006). Glycocalyx disruption is an early marker (Reitsma et al., 2007). Flow-mediated dilation is the most widely used non-invasive functional assessment (Deanfield et al., 2007), though its incremental prognostic value over conventional risk factors is modest.

#### 5.5.2 Impaired vasodilatory function

Endothelial dysfunction is defined by reduced NO bioavailability (Vanhoutte et al., 2017). eNOS uncoupling—driven by BH4 oxidation, ADMA accumulation, arginase-mediated L-arginine depletion, and reductase domain S-glutathionylation—converts a vasoprotective enzyme into a superoxide source (Förstermann and Münzel, 2006). Superoxide reacts with NO at near diffusion-limited rates to generate peroxynitrite (ONOO^−^), which produces lipid peroxidation, protein nitration, and DNA damage (Pacher et al., 2007). Increased endothelin-1 production further compromises vasodilation (Barton and Yanagisawa, 2008).

#### 5.5.3 Microvascular disease

The microvasculature has limited autoregulatory capacity and depends heavily on the endothelial mechanisms described in Section 5.5.2. Hyperglycemia-driven pathways (polyol, AGE, PKC, hexosamine) converge on oxidative stress and inflammation (Dronavalli et al., 2008). Pericyte loss disrupts microvessel integrity via PDGF-B/PDGFRβ and angiopoietin/Tie2 signaling (Armulik et al., 2011). The downstream consequence—microvascular rarefaction and chronic tissue hypoxia—drives the AKI-to-CKD transition and tubulointerstitial fibrosis (Tanaka et al., 2014).

## 6 Therapeutic strategies

**FIGURE 4:**
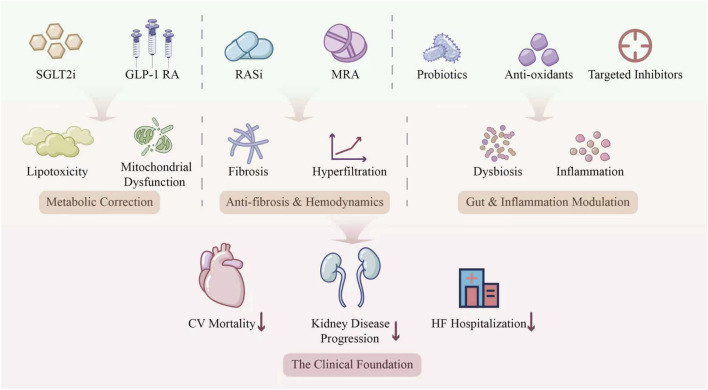
Three mechanism-based therapeutic pathways and clinical endpoints in CKD–CVD comorbidity. This figure groups validated and investigational therapeutic strategies into three parallel mechanism-based pathways (Global and regional, 2020). Metabolic Correction: SGLT2 inhibitors and GLP-1 receptor agonists exert cardiorenal protection by counteracting lipotoxicity and mitochondrial dysfunction (Go et al., 2004). Anti-fibrosis and Hemodynamic Modulation: RAS inhibitors (ACEIs/ARBs) and mineralocorticoid receptor antagonists (MRAs—including the non-steroidal selective MRA finerenone) act by inhibiting fibrosis and reducing glomerular hyperfiltration (Sarnak et al., 2003). Gut and Inflammation Modulation: Probiotics, antioxidants, and targeted inhibitors (e.g., NLRP3 inhibitors, TMA-lyase inhibitors) address dysbiosis and chronic inflammation—this pathway remains supported predominantly by preclinical and early-phase clinical evidence. The three pathways converge at the Clinical Foundation tier and collectively reduce cardiovascular mortality, slow kidney disease progression, and decrease heart failure hospitalization. Panel 4 emphasizes mechanism-based grouping; the complementary evidence-based stratification is shown in Figure 5. Abbreviations: SGLT2i, sodium–glucose cotransporter 2 inhibitor; GLP-1 RA, glucagon-like peptide-1 receptor agonist; RASi, renin–angiotensin system inhibitor; MRA, mineralocorticoid receptor antagonist; CV, cardiovascular; HF, heart failure.

**FIGURE 5:**
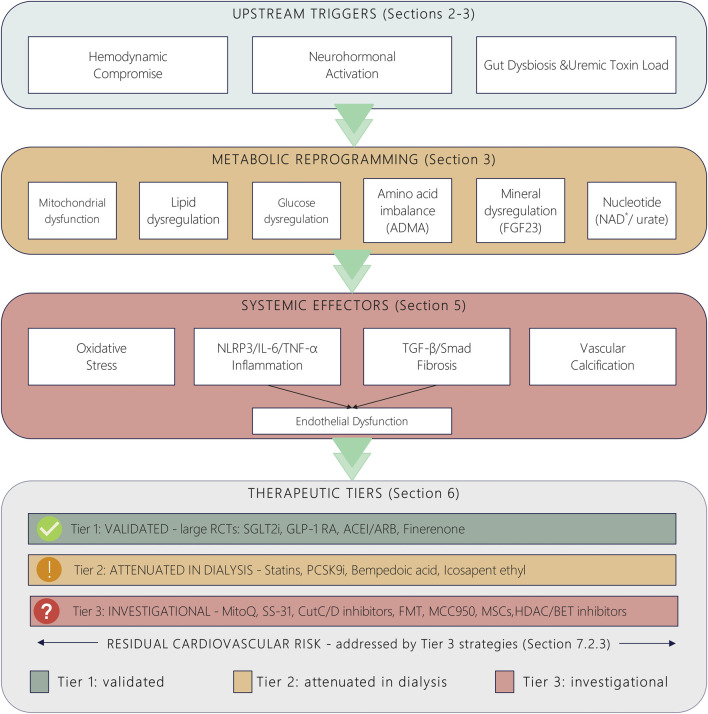
Integrated framework of metabolic reprogramming in CKD–CVD and tiered therapeutic evidence. This figure provides a comprehensive overview of the review and is organized in four hierarchical layers (Global and regional, 2020). Upstream Triggers (Sections 2, 3): hemodynamic compromise, neurohormonal activation, and gut dysbiosis with uremic toxin load (Go et al., 2004). Metabolic Reprogramming (Section 3): mitochondrial dysfunction, lipid dysregulation, glucose dysregulation, amino acid imbalance (represented by ADMA), mineral dysregulation (represented by FGF23), and nucleotide metabolism disturbance (NAD^+^/urate) (Sarnak et al., 2003). Systemic Effectors (Section 5): oxidative stress, NLRP3/IL-6/TNF-α-mediated inflammation, TGF-β/Smad-mediated fibrosis, and vascular calcification—converging on endothelial dysfunction (Weiner et al., 2004). Therapeutic Tiers (Section 6): • Tier 1 (Validated, green): confirmed cardiorenal benefit in large randomized outcome trials—SGLT2i, GLP-1 RA, ACEI/ARB, and finerenone. • Tier 2 (Attenuated in dialysis or limited dedicated CKD evidence, orange): statins (efficacy attenuated in dialysis populations), PCSK9 inhibitors, bempedoic acid, and icosapent ethyl (cardiovascular benefit in general populations but limited dedicated CKD evidence). • Tier 3 (Investigational, red): mechanistically attractive but lacking hard-endpoint validation—MitoQ, SS-31 (elamipretide), CutC/D inhibitors, FMT, MCC950, MSCs, and HDAC/BET inhibitors. The bottom bidirectional arrow emphasizes that residual cardiovascular risk in CKD–CVD is largely driven by pathological domains underaddressed by current standard-of-care and targeted by Tier 3 strategies (see Section 7.2.3). Abbreviations: ADMA, asymmetric dimethylarginine; FGF23, fibroblast growth factor 23; NAD^+^, nicotinamide adenine dinucleotide; NLRP3, NLR family pyrin domain containing 3; IL-6, interleukin-6; TNF-α, tumor necrosis factor alpha; TGF-β, transforming growth factor beta; Smad, mothers against decapentaplegic homolog; SGLT2i, SGLT2 inhibitor; GLP-1 RA, glucagon-like peptide-1 receptor agonist; ACEI, angiotensin-converting enzyme inhibitor; ARB, angiotensin receptor blocker; PCSK9i, proprotein convertase subtilisin/kexin type 9 inhibitor; MitoQ, mitoquinone (mitochondria-targeted coenzyme Q); SS-31, Szeto–Schiller peptide 31/elamipretide (mitochondria-targeted tetrapeptide); CutC/D, choline trimethylamine-lyase; FMT, fecal microbiota transplantation; MCC950, a selective small-molecule NLRP3 inhibitor; MSCs, mesenchymal stem cells; HDAC, histone deacetylase; BET, bromodomain and extraterminal domain protein; RCT, randomized controlled trial.

The therapeutic landscape for CKD–CVD comorbidity has shifted substantially over the past decade. This section organizes available interventions according to the strength of clinical evidence rather than mechanism alone. We adopt a three-tier framework: Tier 1 — interventions with established cardiorenal benefit in large randomized outcome trials; Tier 2 — interventions with cardiovascular outcome data but limited dedicated CKD evidence; and Tier 3 — investigational strategies supported primarily by mechanistic studies, biomarker endpoints, or early-phase trials. Figure 4 summarizes the mechanism-based therapeutic pathways, and Figure 5 the corresponding evidence-tier framework.

### 6.1 Glucose metabolism—validated cornerstones

#### 6.1.1 SGLT2 inhibitors (tier 1)

SGLT2 inhibitors are the most robustly validated metabolic intervention in CKD–CVD. Beyond glycemic control, they deliver cardiorenal protection through multiple glucose-independent mechanisms (Heerspink et al., 2020; Vallon and Thomson, 2020).

##### 6.1.1.1 Mechanisms

Reduced proximal tubular sodium reabsorption increases distal sodium delivery, restoring tubuloglomerular feedback through adenosine-mediated afferent arteriolar vasoconstriction (Girardi et al., 2024). The result is lowered intraglomerular pressure, reduced hyperfiltration, and natriuretic-osmotic preload reduction without the paradoxical RAAS activation seen with loop diuretics (Girardi et al., 2024). By reducing proximal tubular workload, SGLT2 inhibitors decrease tubular oxygen demand by 20%–30%, relieving the chronic hypoxic stress that drives tubulointerstitial fibrosis (Layton et al., 2016). They also promote hepatic ketogenesis, providing β-hydroxybutyrate as an oxygen-efficient cardiac fuel, and inhibit cardiomyocyte NHE1, attenuating sodium–calcium overload (Piperis et al., 2024). E-LIFT showed empagliflozin reduces hepatic fat in T2DM with NAFLD (Kuchay et al., 2018).

##### 6.1.1.2 Clinical evidence

EMPA-REG OUTCOME (n = 7,020) demonstrated 38% reduction in cardiovascular death, 35% reduction in heart failure hospitalization, and 32% reduction in all-cause mortality with empagliflozin in type 2 diabetes with established CVD (26). DAPA-HF confirmed dapagliflozin’s benefit in HFrEF independent of diabetes status (McMurray et al., 2019), and EMPEROR-Reduced corroborated this in patients with reduced ejection fraction (Packer et al., 2020). DAPA-CKD (n = 4,304) showed dapagliflozin reduces renal composite endpoints by 39% in CKD with or without diabetes (Heerspink et al., 2020).

Despite the strength of the evidence base, several boundaries deserve recognition. Benefits attenuate at very low eGFR (<25 mL/min/1.73 m^2^), with limited data in dialysis populations. Subgroup heterogeneity exists across ethnicity, HFpEF *versus* HFrEF, and presence or absence of albuminuria. Real-world adoption is constrained by diabetic ketoacidosis (especially during acute illness or perioperatively), genitourinary infections, and volume depletion in elderly patients on diuretics. The position of SGLT2 inhibitors as foundational therapy is empirically secure, but the precision-medicine question of *which* CKD–CVD patient benefits *most* remains open.

#### 6.1.2 GLP-1 receptor agonists (tier 1)

GLP-1 receptor agonists enhance glucose-dependent insulin secretion, suppress glucagon, delay gastric emptying, and reduce appetite (Drucker, 2018). GLP-1 receptors are widely distributed across the pancreas, heart, kidney, and vascular endothelium. Significant weight loss further improves insulin sensitivity, blood pressure, and lipid profile.

##### 6.1.2.1 Clinical evidence

LEADER (n = 9,340) showed liraglutide reduces MACE by 13% and cardiovascular mortality by 22% (Marso et al., 2016); the renal composite endpoint was reduced by 22% (Mann et al., 2017). REWIND demonstrated dulaglutide reduces MACE by 12% (Gerstein et al., 2019). More recent agents (semaglutide, tirzepatide) have extended these benefits.

Although cardiovascular outcomes are robust, several caveats apply. Gastrointestinal adverse events are common and limit tolerability. Rare cases of pancreatitis and gallbladder disease have been reported. Injectable formulations limit broader adoption. Dedicated trials in advanced CKD remain limited, although the recent FLOW trial (semaglutide in CKD) has begun to fill this gap.

### 6.2 Lipid metabolism—tier one in general risk; attenuated in dialysis

Statins inhibit HMG-CoA reductase, reduce hepatic cholesterol, and upregulate LDL receptor expression to lower LDL-C (Grundy et al., 2019; Collins et al., 2016). They also exert pleiotropic effects on endothelial function and plaque stability.

#### 6.2.1 Critical appraisal—statins in CKD

SHARP (simvastatin plus ezetimibe, n = 9,270) reduced major atherosclerotic events by 17% in CKD patients (Baigent et al., 2011). However, the 4D trial (atorvastatin in hemodialysis diabetics) and AURORA (rosuvastatin in dialysis) failed to demonstrate cardiovascular benefit. Statin efficacy diminishes with advancing renal disease, and uremia-modified lipoproteins (carbamylated LDL, oxidized lipids—see Section 3) may explain this attenuation. In dialysis-dependent patients, routine statin initiation is no longer recommended, although continuation in patients already on therapy remains reasonable.

#### 6.2.2 PCSK9 inhibitors (tier 2)

PCSK9 binds hepatic LDL receptors and accelerates their degradation (Seidah et al., 2014). Evolocumab plus statin reduced cardiovascular events by 15% in FOURIER (Sabatine et al., 2017). Inclisiran lowers LDL-C through siRNA-mediated PCSK9 silencing with twice-yearly injection, simplifying compliance (Ray et al., 2020) (26). Dedicated outcome data in CKD remain limited, though post-hoc analyses suggest preserved efficacy across eGFR strata.

#### 6.2.3 Bempedoic acid (tier 2)

This ATP citrate lyase inhibitor acts upstream of HMG-CoA reductase and is hepatically activated, reducing muscle-related toxicity. CLEAR Outcomes (n = 13,970) showed 13% reduction in major cardiovascular events in statin-intolerant patients (Nissen et al., 2023).

#### 6.2.4 Icosapent ethyl (tier 2)

REDUCE-IT showed 25% reduction in cardiovascular events with high-dose purified eicosapentaenoic acid in patients with hypertriglyceridemia (Bhatt et al., 2019). The STRENGTH trial of mixed omega-3, however, was negative—suggesting that formulation specificity matters and broader generalization is unwarranted.

### 6.3 Energy metabolism—tier 3 (investigational)

This section presents interventions with mechanistic appeal but limited clinical confirmation in CKD–CVD.

#### 6.3.1 Coenzyme Q10

CoQ10 is depleted in failing myocardium. Q-SYMBIO (n = 420) reported reduced major adverse cardiovascular events with CoQ10 supplementation in chronic heart failure (Mortensen et al., 2014). Q-SYMBIO was a single, modest-sized trial. Broader meta-analyses have produced inconsistent results, and CoQ10 has not been validated as standard heart failure therapy. Dedicated CKD trials are absent.

#### 6.3.2 MitoQ and other mitochondria-targeted antioxidants

MitoQ accumulates in mitochondria via triphenylphosphonium conjugation and reduces oxidative stress in animal and small human studies (Smith and Murphy, 2010). However, larger trials of related agents—elamipretide in EMBRACE-STEMI and PROGRESS-HF—failed to meet primary endpoints. These approaches remain investigational.

#### 6.3.3 Substrate modulators

Trimetazidine inhibits mitochondrial 3-ketoacyl-CoA thiolase, shifting cardiac metabolism toward glucose oxidation and improving oxygen efficiency (Kantor et al., 2000). Despite mechanistic appeal, trimetazidine is not approved in many regions, and post-marketing concerns about parkinsonism have limited adoption. Ranolazine inhibits late sodium current with anti-anginal effects (Banon et al., 2014); its use in CKD–CVD beyond refractory angina is not established.

### 6.4 Renin–angiotensin–aldosterone system inhibition—validated cornerstone

ACE inhibitors and ARBs lower blood pressure, reduce proteinuria, and slow CKD progression. Beyond hemodynamic effects, they exert direct metabolic actions: ACE inhibition partially restores PGC-1α expression and mitochondrial biogenesis in pressure-overloaded cardiomyocytes (de Cavanagh et al., 2015), reversing aspects of the metabolic reprogramming described in Sections 3–5. RAAS inhibitors are first-line therapy for proteinuric CKD, with monitoring required for hyperkalemia and acute renal injury.

#### 6.4.1 Mineralocorticoid receptor antagonists

Aldosterone is pro-fibrotic and pro-inflammatory beyond sodium retention. RALES showed spironolactone reduces morbidity and mortality in severe heart failure (Pitt et al., 1999). Hyperkalemia limits steroidal MRA use in CKD. Finerenone—a non-steroidal selective MRA with lower hyperkalemia risk—improved cardiorenal outcomes in FIDELIO-DKD (Bakris et al., 2020). The pooled FIDELITY analysis (FIDELIO-DKD plus FIGARO-DKD) confirmed benefit across the diabetic CKD spectrum, establishing finerenone as a Tier one agent in this indication.

### 6.5 Lifestyle interventions

Low-protein diet is recommended in non-dialysis CKD stages 3–5 to reduce uremic toxin generation and hyperfiltration, with monitoring to prevent malnutrition (Ikizler et al., 2020). The Mediterranean dietary pattern improves cholesterol efflux gene expression and reduces cardiovascular events in high-risk populations (Hernando-Redondo et al., 2025). Regular aerobic and resistance exercise improves insulin sensitivity, blood pressure, lipids, and mitochondrial biogenesis. Dedicated trials of dietary patterns in CKD–CVD are limited, and adherence in real-world settings is often suboptimal. Exercise remains underutilized in CKD populations due to fatigue, anemia, and cardiovascular comorbidities.

### 6.6 Emerging therapeutic strategies (tier 3)

These approaches address pathways underserved by current standard-of-care but lack large outcome trial validation.

#### 6.6.1 Gut microbiota targeting

Probiotic supplementation reduces LPS levels and improves cardiometabolic markers in obese postmenopausal women (Szulińska et al., 2018). Fecal microbiota transplantation from lean donors transiently improves insulin sensitivity in metabolic syndrome (Vrieze et al., 2012). TMA-lyase inhibitors—targeting microbial enzymes rather than host physiology—have shown preclinical efficacy in reducing TMAO and thrombosis (Witkowski et al., 2022).

Probiotics, FMT, and TMA-lyase inhibitors all remain at proof-of-concept or early-phase clinical stages. No large RCT has demonstrated hard renal or cardiovascular endpoints. Microbiome-based therapeutics face unique regulatory challenges related to donor variability, manufacturing standardization, and durability of effect. These strategies address a pathway largely untouched by guideline-directed therapy but should not be deployed as if validated.

#### 6.6.2 Gene and epigenetic therapies

Circulating miRNA profiles are altered in metabolic disease (Seyhan et al., 2016), and antagomirs/mimics are under preclinical investigation. Delivery efficiency, off-target effects, and long-term safety remain major obstacles. Epigenetic mechanisms in diabetic vascular complications are well established, but bromodomain inhibitors and HDAC inhibitors have not yet entered cardiorenal clinical use (Reddy and Natarajan, 2011).

#### 6.6.3 Cell therapy

Mesenchymal stem cells (MSCs) are under investigation for cardiac repair and AKI recovery (Squillaro et al., 2016). MSC-derived exosomes may mediate stem cell therapeutic effects with greater safety and reproducibility (Kalluri and LeBleu, 2020).

Most published MSC trials in cardiorenal disease are small phase I/II studies with inconsistent outcomes—examples include POSEIDON and C-CURE, which produced null or modest results on hard endpoints. Manufacturing standardization, donor variability, dose, and route of administration remain unresolved. For exosome therapeutics, fewer than 20% of published preclinical reports fully comply with MISEV reporting standards, raising reproducibility concerns. MSC- and exosome-based strategies for CKD–CVD remain investigational; definitive evidence is at least a decade away.

#### 6.6.4 Section synthesis

Among the strategies discussed, only SGLT2 inhibitors, GLP-1 receptor agonists, RAAS inhibitors, and the non-steroidal selective MRA finerenone have established benefit in CKD–CVD outcome trials. Lipid-lowering therapies are validated for general cardiovascular prevention but show attenuated efficacy in dialysis. Energy metabolism, microbiome, gene, and cell-based interventions remain mechanistically appealing but clinically unproven. The therapeutic enthusiasm appropriate for SGLT2 inhibitors should not be extended uncritically to investigational strategies—a distinction we have endeavored to maintain throughout this review.

## 7 Perspectives

### 7.1 Current research limitations

Despite substantial mechanistic progress, several limitations constrain translation to clinical practice.

First, the gap between basic science and clinical application remains wide. Animal models—predominantly rodent ischemia-reperfusion, 5/6-nephrectomy, and adenine-induced CKD—capture isolated dimensions of human pathology but fail to recapitulate the chronic, multi-system reprogramming that defines CKD–CVD comorbidity. The Global Kidney Health Initiative has formally identified bridging this gap as a research priority (Levin et al., 2017).

Second, CKD patients are highly heterogeneous in genetic background, disease severity, comorbidity profile, and therapeutic response (Webster et al., 2017). This heterogeneity confounds trial design, attenuates treatment effects in unselected populations, and limits generalizability of subgroup findings.

Third, metabolic pathways operate as networks rather than isolated targets. Combined epidemiologic and metabolomic approaches predict CKD progression better than either alone (Rhee et al., 2013), but multi-pathway integration remains methodologically immature. Single-pathway pharmacology cannot fully address a system in which lipid metabolism, oxidative stress, inflammation, fibrosis, and Nrf2 dysfunction interact bidirectionally.

Fourth, the literature has a publication bias toward positive mechanistic findings. The repeated failure of broad-spectrum antioxidant interventions (HOPE, BEACON-bardoxolone), the negative AST-120 trials in CKD progression, the disappointing trimetazidine post-marketing experience, and the inconsistent MSC outcomes (POSEIDON, C-CURE) collectively delineate a translational chasm between mechanistic plausibility and therapeutic utility. These failures share a common pattern—broad-spectrum mechanistic targeting in unselected populations. Future trials should incorporate metabolic-phenotype stratification at enrollment, target-engagement biomarkers as inclusion criteria, and hard renal/cardiovascular endpoints rather than surrogate markers. Future reviews should give equal weight to negative trials.

### 7.2 Future research directions

#### 7.2.1 Precision medicine

Precision medicine in CKD–CVD will depend on operational stratification rather than aspirational labeling. Diabetic kidney disease research has begun to define molecular subtypes amenable to targeted intervention (Tuttle et al., 2022). Pharmacogenomic variation is one tractable dimension: polymorphisms in OCT1 modulate metformin pharmacokinetics and inform genotype-based prescribing (Shu et al., 2008). Whether comparable stratification will deliver meaningful clinical benefit for SGLT2 inhibitors, GLP-1 receptor agonists, or finerenone remains to be tested prospectively.

A practical near-term goal is identification of patients most likely to respond to a given metabolic intervention—distinguishing, for example, those whose residual cardiovascular risk derives primarily from inflammation (candidates for inflammasome targeting) *versus* those whose risk reflects mitochondrial dysfunction or microbiome dysbiosis. This requires integration of metabolomics, transcriptomics, and clinical phenotype data with prospective treatment-response endpoints.

#### 7.2.2 Multi-omics integration

Genomics, transcriptomics, proteomics, and metabolomics offer complementary information layers. Combining these layers can identify regulatory nodes invisible to single-omics analysis and may reveal druggable targets that link genotype to clinical phenotype (Hasin et al., 2017). Machine learning enables automated feature extraction and pattern recognition from these high-dimensional datasets (Libbrecht and Noble, 2015).

Multi-omics integration is at present a methodological aspiration more than a clinical reality. Most published multi-omics CKD studies are cross-sectional, single-center, and underpowered. Reproducibility across cohorts is limited by analytical pipeline heterogeneity, batch effects, and inadequate sample sizes. For multi-omics to deliver clinically actionable stratification, the field requires harmonized protocols, large prospective cohorts, and validated computational pipelines—none of which is presently mature.

#### 7.2.3 Bridging residual risk: underaddressed pathological domains

Despite optimal statin therapy, RAAS blockade, and glycemic control, CKD–CVD patients retain substantial residual cardiovascular risk attributable to four pathological domains underaddressed by current standard-of-care.

##### 7.2.3.1 Mitochondrial bioenergetic failure

Existing therapies do not restore proximal tubular fatty acid oxidation or correct the mitochondrial energetic deficit that drives tubular atrophy and pharmacokinetic vulnerability (Doke and Susztak, 2022). Mitochondria-targeted antioxidants (SS-31/elamipretide) and NAD^+^ precursors are under preclinical and early clinical evaluation, although confirmatory trials remain outstanding.

##### 7.2.3.2 Protein-bound uremic toxin accumulation

Indoxyl sulfate and p-cresyl sulfate are more than 90% protein-bound and incompletely removed by hemodialysis. The organ-level consequences are well characterized (Lowenstein and Nigam, 2021). Upstream gut microbiota-targeted approaches—microbial enzyme inhibitors, prebiotic fiber, and intestinal barrier restoration—are complementary to dialytic clearance but require large outcome trials.

##### 7.2.3.3 NLRP3 inflammasome activation

The CANTOS trial established proof-of-concept that IL-1β blockade with canakinumab reduces major adverse cardiovascular events in high-inflammatory-risk patients, including those with impaired renal function (Ridker et al., 2017). However, IL-18 and IL-6 remain elevated following IL-1β inhibition, indicating residual inflammatory risk and supporting NLRP3 itself as a more complete therapeutic target (Ridker et al., 2020). The selective NLRP3 inhibitor MCC950 reduced atherosclerotic plaque burden and macrophage pyroptosis in ApoE^−/−^ mice without compromising systemic immunity (Zeng et al., 2021). Recent reviews identify MCC950 derivatives as the most advanced NLRP3-targeting candidates for clinical translation (Zheng et al., 2024). Whether NLRP3 inhibition can replicate canakinumab’s modest absolute benefit while avoiding its infection-related harms is the central translational question.

##### 7.2.3.4 Epigenetic metabolic memory

Hyperglycemia and uremic toxin exposure induce persistent pro-inflammatory epigenetic modifications, including acetylation changes at NF-κB target loci. These modifications survive metabolic correction and constitute a residual inflammatory substrate refractory to current pharmacotherapy. HDAC and BET inhibitors are under investigation as epigenetic erasers, although their cardiovascular and renal applications remain investigational.

Systematically addressing these four domains is more likely to reduce residual cardiovascular mortality than further intensification of conventional risk factor modification. SGLT2 inhibitors, validated in CREDENCE (Perkovic et al., 2019) and DAPA-CKD (Heerspink et al., 2020), illustrate that mechanism-driven metabolic interventions can deliver clinical benefit. The challenge is to extend this success to currently underserved pathological domains through rationally designed combination strategies that reflect the network nature of CKD–CVD pathology.

#### 7.2.4 Prevention strategies

Prevention should focus on early intervention before metabolic dysregulation produces irreversible structural injury. Risk prediction models that integrate clinical risk factors with metabolic biomarkers can identify high-risk individuals at earlier disease stages (Gansevoort et al., 2013). Lifestyle interventions—Mediterranean dietary pattern, regular aerobic and resistance exercise—have demonstrated efficacy in primary prevention but remain underutilized. Digital health tools, including wearable devices and mHealth applications, may enable scalable implementation and remote monitoring. The most cost-effective prevention strategy may be aggressive identification and treatment of CKD at stages G1–G2, when albuminuria-mediated cardiovascular risk is already elevated but tubulointerstitial damage is potentially reversible.

## 8 Conclusion

The pathophysiology of CKD–CVD comorbidity is dominated by abnormal metabolism. Metabolic disturbances are not merely consequences of disease; they actively contribute to its progression. This review has summarized disordered energy, lipid, glucose, amino acid, mineral, and nucleotide metabolism in the cardiorenal axis, together with their gut–microbiota, oxidative, inflammatory, fibrotic, calcific, and endothelial systemic effectors.

Among the strategies discussed, the evidence base divides clearly into three tiers. Validated interventions—SGLT2 inhibitors, GLP-1 receptor agonists, RAAS inhibitors, and the non-steroidal selective MRA finerenone—have established cardiorenal benefit in large outcome trials. Attenuated interventions—statins and other lipid-lowering therapies—retain value in general cardiovascular prevention but show diminished efficacy in dialysis populations. Unproven interventions—mitochondria-targeted antioxidants, gut microbiota modulation, epigenetic therapy, gene-based and cell-based approaches—are mechanistically appealing but clinically unvalidated. Their inclusion in this review reflects scientific potential, not demonstrated benefit.

The path forward requires three commitments. First, rigorous evidence stratification that distinguishes validated from investigational therapies and resists the conflation of mechanistic plausibility with clinical efficacy. Second, honest engagement with negative trials that delineate the boundaries of mechanistic enthusiasm and refine future hypothesis generation. Third, multidisciplinary collaboration across nephrology, cardiology, endocrinology, molecular biology, and bioinformatics, supported by prospective, adequately powered trials with hard renal and cardiovascular endpoints rather than surrogate biomarkers.

CKD–CVD comorbidity will not be solved by a single agent or a single mechanism. It will be solved, if at all, through sustained and evidence-disciplined integration across the metabolic, microbial, immune, and pharmacokinetic domains outlined in this review.
