# Supplementary material for: Metabolic reprogramming in chronic kidney disease-cardiovascular disease comorbidity: from molecular mechanisms to therapeutic strategies
Source: Front Pharmacol. 2026 Jul 9;17:1801502. doi: 10.3389/fphar.2026.1801502 (PMC13392259; doi:10.3389/fphar.2026.1801502)
Supplement: Supplementary file 1 [file Table1.docx]

**Table**

**Table S1. Summary of evidence tiers for therapeutic strategies in CKD–CVD comorbidity.**

| **Strategy** | **Tier** | **Pivotal trials** | **Hard endpoint** | **CKD-specific evidence** | **Major caveats** |
| --- | --- | --- | --- | --- | --- |
| SGLT2 inhibitors | 1 | EMPA-REG, DAPA-CKD, EMPEROR-Reduced | ✓ CV death, HF hosp, renal | ✓ DAPA-CKD | DKA, GU infections, eGFR <25 |
| GLP-1 RA | 1 | LEADER, REWIND, FLOW | ✓ MACE, renal | Limited advanced CKD | GI tolerance |
| ACEI/ARB | 1 | Multiple legacy | ✓ Renal, BP | ✓ Established | Hyperkalemia |
| Finerenone | 1 | FIDELIO-DKD, FIGARO-DKD | ✓ Renal, CV | ✓ Diabetic CKD only | Hyperkalemia |
| Statins | 2 | SHARP / 4D / AURORA | ✓ CV(non-dialysis); ✗ dialysis | Attenuated in dialysis | Carbamylated LDL |
| PCSK9i | 2 | FOURIER | ✓ CV | Limited dedicated CKD | Cost |
| MitoQ / SS-31 | 3 | EMBRACE-STEMI, PROGRESS-HF (negative) | ✗ | None | Failed in late-phase trials |
| FMT / Probiotics | 3 | Small phase II | ✗ | None | Regulatory uncertainty |
| MCC950 (NLRP3) | 3 | Preclinical only | ✗ | None | Translation pending |
| MSC / Exosomes | 3 | POSEIDON, C-CURE (mixed) | ✗ | None | Standardization gap |
